# Novel Bread Wheat Lines Enriched in Carotenoids Carrying *Hordeum chilense* Chromosome Arms in the *ph1b* Background

**DOI:** 10.1371/journal.pone.0134598

**Published:** 2015-08-04

**Authors:** María-Dolores Rey, María-Carmen Calderón, María Jesús Rodrigo, Lorenzo Zacarías, Enriqueta Alós, Pilar Prieto

**Affiliations:** 1 Departamento de Mejora Genética Vegetal, Instituto de Agricultura Sostenible, Consejo Superior de Investigaciones Científicas, Apartado, Córdoba, Spain; 2 Instituto de Agroquímica y Tecnología de Alimentos, Consejo Superior de Investigaciones Científicas, Paterna, Valencia, Spain; Murdoch University, AUSTRALIA

## Abstract

The use of crop wild relative species to improve major crops performance is well established. *Hordeum chilense* has a high potential as a genetic donor to increase the carotenoid content of wheat. Crosses between the 7**H^ch^** *H*. *chilense* substitution lines in wheat and the wheat *pairing homoeologous1b (ph1b*) mutant allowed the development of wheat-*H*. *chilense* translocation lines for both 7**H^ch^**α and 7**H^ch^**β chromosome arms in the wheat background. These translocation lines were characterized by *in situ* hybridization and using molecular markers. In addition, reverse phase chromatography (HPLC) analysis was carried out to evaluate the carotenoid content and both 7**H^ch^**α∙7**A**L and 7**A**S∙7**H^ch^**β disomic translocation lines. The carotenoid content in 7**H^ch^**α∙7**A**L and 7**A**S∙7**H^ch^**β disomic translocation lines was higher than the wheat-7**H^ch^** addition line and double amount of carotenoids than the wheat itself. A proteomic analysis confirmed that the presence of chromosome 7**H^ch^** introgressions in wheat scarcely altered the proteomic profile of the wheat flour. The *Psy1* (*Phytoene Synthase1*) gene, which is the first committed step in the carotenoid biosynthetic pathway, was also cytogenetically mapped on the 7**H^ch^**α chromosome arm. These new wheat-*H*. *chilense* translocation lines can be used as a powerful tool in wheat breeding programs to enrich the diet in bioactive compounds.

## Introduction

Wild species of bread wheat *Triticum aestivum* (2n = 6x = 42, genome **AABBDD**) are important resources for broadening the genetic variability of crop plants and useful traits have been transferred from these species to wheat [1]. *Hordeum chilense* (2n = 2x = 14, genome **H** ^**ch**^ **H** ^**ch**^) is an extremely polymorphic diploid wild barley from South of America. It has high crossability with other members of the *Triticeae* tribe and presents several agronomical characteristics which could be transferred into wheat, such as high carotenoid content among others [2–6]. *Hordeum chilense* addition and substitution lines in wheat [7–9] are generally used as a bridge to generate wheat-*H*. *chilense* translocation or recombinant lines [10–11]. However, pairing between wheat and related chromosomes from these species is rare [12]. Chromosome pairing between homoeologous (related) chromosomes can be achieved using the *ph1b* mutant [10]. The *Ph1* locus, which is located on the 5**B**L chromosome arm, ensures chromosome pairing and recombination between homologous (identical) chromosomes [13–16]. In the absence of the *Ph1* locus (*pairing homoeologous*1 locus; *ph1b* mutant) unspecific chromosome associations can occur between related chromosomes and therefore can be used to induce homoeologous recombination [17]. An extensive molecular analysis of the region including the *Ph1* locus has been carried out, and the *Ph1* locus has been restricted to a 2.5 Mb region containing a cluster of *Cdk-2 (cyclin dependent kinase-2)* related genes [18], and regulates premeiotic replication, chromatin condensation, transcription of the earliest meiotic gene (*Asy1*), homologue pairing/synapsis, resolution of incorrect pairing at pachytene and recombination [19–21].

The *ph1b* mutant can be used to facilitate interspecific recombination between chromosomes from wheat and those chromosomes from related species to transfer desirable agronomics traits from those relatives into wheat [22]. For example, bread wheat has lower carotenoid contents than other plant species [23]. Carotenoids are a diverse family of natural isoprenoid pigments responsible of the characteristic color, from pale yellow to red, of different plant tissues and organs [24]. Carotenoids play crucial roles in many plant physiological processes and are essential for animals since some of them are the precursors of vitamin A and have a broad range of function, as antioxidants and other health-related properties [25]. Since carotenoids are almost exclusively synthetized by plants, and certain fungi and bacteria, animals and humans rely upon the diet as the source of these compounds. Carotenoids can be grouped in two main classes: carotenes, which are tetraterpenoid hydrocarbons, and xanthophylls, which are carotenoids with one or more oxygenated groups in the molecule. Lutein, a xanthophyll which accumulates in eye macula and plays an essential role in human vision, is the main carotenoid found in wheat, and is, in most cases, accompanied by lower amounts of zeaxanthin, β-cryptoxanthin and β-carotene [26–29]. The chromosomal location of genes involved in carotenoid synthesis in *H*. *chilense* was deciphered using *H*. *chilense* addition lines in wheat [2]. The presence of chromosome 7**H** ^**ch**^ of *H*. *chilense* increased the carotenoid content in wheat, and moreover, the ditelosomic addition line for 7**H** ^**ch**^α chromosome arm showed greater influence on the pigment content [2]. A chromosomal region on the distal part of chromosome 7**H** ^**ch**^β of *H*. *chilense* related to the carotenoid content has been recently reported [30]. New genes controlling the carotenoid content were also found in the genome of *H*. *chilense*, such as *Carot1* and *Zds* (codifying for a zeta-carotene desaturase) genes, located on the centromeric region of chromosome 2**H** ^**ch**^ and the *Psy1* (*Phytoene synthase 1*) gene, which was located on the 7**H** ^**ch**^ *α* chromosome arm [30–32]. In fact, the enzyme PSY catalyses the first step of the carotenoids biosynthetic pathway and it is considered a limiting factor for carotenoid production [33].

Genomic *in situ* hybridization (GISH) is the most efficient and accurate technique to estimate the amount of alien chromatin introgressed in wheat [34]. Moreover, fluorescence *in situ* hybridization (FISH) combined with GISH enables the determination of the exact chromosomal compositions and resolutions of the chromosome arms involved in wheat-*H*. *chilense* translocations [35]. *In situ* hybridization can be also used to physically map single-copy genes on mitotic chromosomes [36].

Classical genetic breeding can result in undesirable side-effects as a consequence of the alteration of the genomic composition. Thus it is important to evaluate the quality of the introgression lines produced by conventional breeding. Ten to fifteen percent of the wheat grain dry weight are proteins, mainly storage proteins, which are the major responsible of dough properties, and also other minority proteins which might modify flour quality and/or be involved in hypersensitivity reactions such as food allergy and celiac disease [37–42]. Hence, deciphering the composition of the endosperm proteins through proteomics approaches is useful to evaluate the potential interest of wheat introgression lines.

In this paper, we describe the development and characterization of new wheat-*H*. *chilense* translocation lines for both 7**H** ^**ch**^α and 7**H** ^**ch**^β chromosome arms with the aim of increasing the wheat carotenoid content. In addition, the *Psy1* gene, the first committed step in the carotenoid biosynthetic pathway, was cytogenetically mapped on *H*. *chilense* chromosome 7**H** ^**ch**^. The study is supplemented by an analysis of the proteomic profile of the flour of these new wheat-*H*. *chilense* translocation lines with a higher carotene content.

## Material and Methods

### Plant material

**Fig 1:**
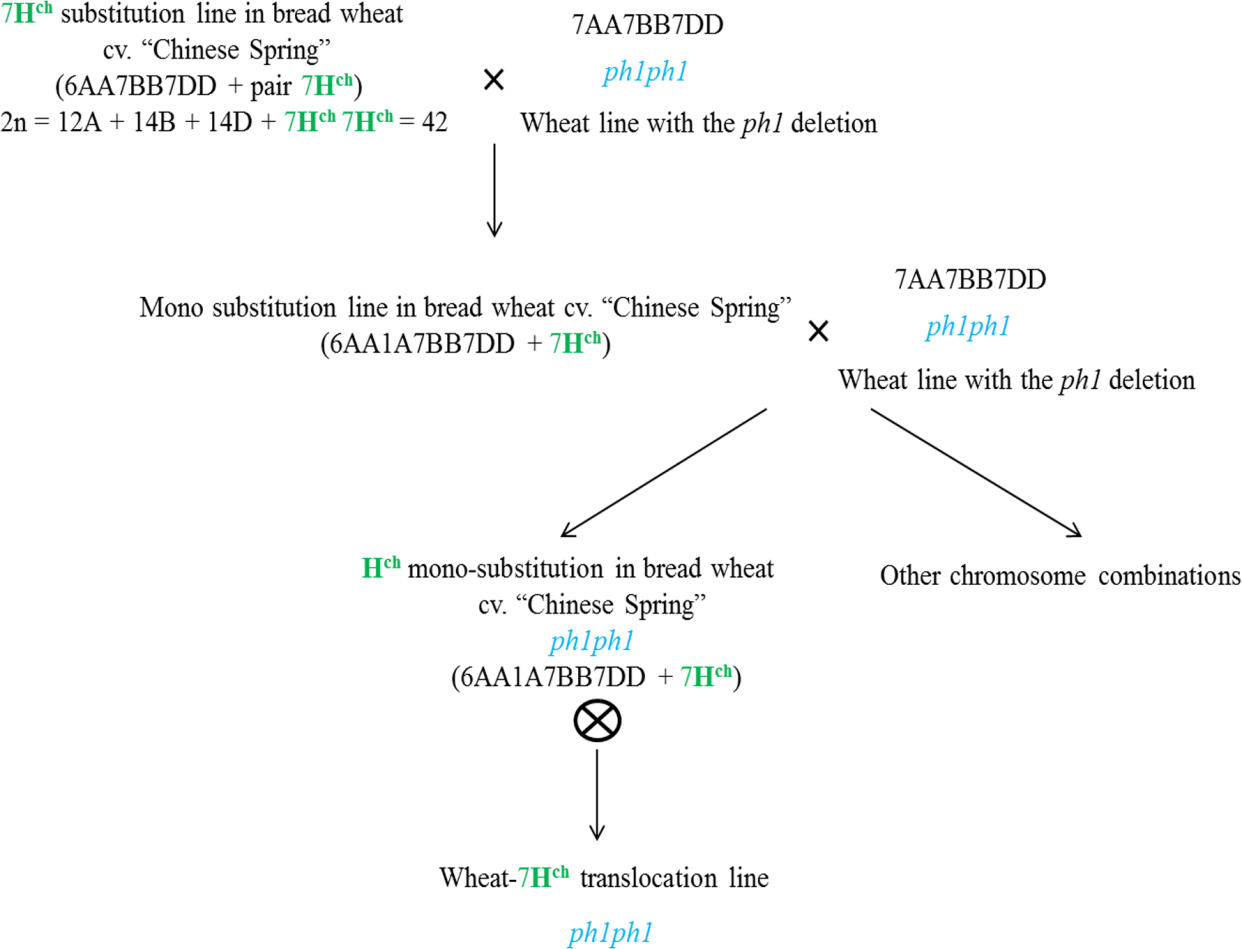
Development of *H*. *chilense* introgression lines in hexaploid wheat in the *ph1b* mutant background. Crosses between the 7**H** ^**ch**^ substitution line in bread wheat and the *ph1b* mutant were developed and backcrossed to the *ph1b* mutant to obtain *Hordeum* translocation in the absence of the *Ph1* locus.

*Hordeum chilense* substitution lines for chromosome 7**H** ^**ch**^ in bread wheat [7] were used as parental lines in initial crosses with the wheat line deficient for the *Ph1* locus (*Triticum aestivum* cv. *`*Chinese Spring´ (CS), *ph1bph1b* genotype; [22, 43]). The descendence was backcrossed by the wheat *ph1b* mutant to obtain chromosome 7**H** ^**ch**^ in the *ph1b* mutant background as described in Fig 1. Seeds from the descendence of the backcrosses were germinated in Petri dishes on wet filter papers in darkness for 5 days at 4°C followed by 24 hours incubation at 25°C. Roots about 1 cm long were cut, incubated for 4 hours in a 0.05% colchicine solution at 25°C and then fixed in 100% ethanol- acetic acid, 3:1 (v/v). Fixed roots were stored at 4°C for at least 1 month to perform cytogenetic experiments. All plants were grown in a greenhouse at 26°C (day) and 22°C at night with a photoperiod of long days (16 h of daylight—8 h of darkness).

### Characterization of the translocation lines using molecular markers

Genomic DNA was extracted from frozen young leaf tissue using the cetyltrimethylammonium bromide (CTAB) method [44] with some modifications according to [45]. Bread wheat *ph1b* mutants were checked for the *ph1b* deletion using the ABC_920_ SCAR marker as previously described [46]. The PCR reactions were performed in 30 μl of reaction mixture containing 1x PCR buffer with MgCl_2_ (Bioline USA, Taunton, MA), 0.25 mM dNTPs, 5 pmol primers, 0.02 U/ μl of Taq DNA polymerase (Bioline USA, Taunton, MA). The PCR cocktail was initially denatured at 94°C for 5 min, and then the amplification reaction consisted in 35 cycles of 1 min at 94°C, 1 min at 51°C and 1 min at 72°C, followed by a final extension reaction of 7 min at 72°C. The PCR products were solved on 1% agarose gels in 1xTBE and visualized by ethidium bromide staining under UV light. The presence of both 7**H** ^**ch**^α and 7**H** ^**ch**^β chromosome arms was analyzed using the microsatellites BAWU550 and BAWU763, respectively, as described in [47]. The confirmation of the wheat chromosome involved in chromosome translocations (chromosome 7**A**) was also carried out using the microsatellites Xgwm471-7**A**S, Xgwm332-7**A**L as described in [48].

### Cytogenetic analysis

GISH experiments were performed according to [49] using genomic *H*. *chilense* DNA as probe to confirm the presence of chromosome 7**H** ^**ch**^. Sonicated salmon sperm DNA was used as blocking DNA (salmon sperm DNA: DNA probe, 2:1). The identification of the 7**H** ^**ch**^α or 7**H** ^**ch**^β chromosomes arms was also confirmed by FISH using the pAs1 sequences [50–51]. The wheat chromosome arms involved in inter-specific translocations with the *H*. *chilense* chromosome 7**H** ^**ch**^ were also identified using both the GAA-satellite sequence [52–53] and the pAs1 probe [51] as described in [54].

### Physical mapping of *Psy1*

Physical localization of *Psy1* gene from the carotenoid biosynthetic pathway was performed by *in situ* hybridization. A 2538bp genomic region of the *Psy1* gene was amplified by PCR in a (7**A**)7**H** ^**ch**^ substitution line in bread wheat to be used as a probe in further *in situ* hybridization experiments. A pair of primers was designed using the Primer3plus software [55] based on the *Psy1* sequence previously described in *H*. *chilense* (GenBank accession number HM598415) [32, 56–57]. The sequences for the forward and reverse primers used for *Psy1* amplification were, 5’AGTGGTGAATCCATCCCTTG3’ and 5’CCTTCCTCTTCTTGCACTGG3’, respectively. PCR amplification for *Psy1* gene was performed using MyFi DNA polymerase (Bioline USA, Taunton, MA) according to the manufacturer´s instructions as follows: 3 min 94°C, 35 cycles of 15 s at 94°C, 15 s at 60°C and 3.5 min at 72°C. PCR products were resolved on 1% agarose gels in 1xTBE and stained with ethidium bromide and visualized under UV light. The PCR fragments corresponding to the *Pys1* locus amplified from both *H*. *chilense* (used as a positive control) and the (7**A**)7**H** ^**ch**^ substitution line, were sequenced to confirm the identity of the gene probe.

Chromosome spreads from root tips of germinated wheat seeds, probe labelling and *in situ* hybridization were carried as described by [35]. Detection of hybridization signals was carried out using the Tyramide Signal Amplification Kit (TSA, PerkinElmer Life and Analytical Sciences, Inc., Waltham, MA, USA). To identify wheat chromosomes with positive signals, samples were re-hybridized using the pAs1 repetitive sequence and GAA-satellite sequence as probes [51–52]. Individual slides were observed under a Nikon Eclipse 80i, microscope (Nikon Instruments Europe BV, UK). Images were captured with a Nikon CCD camera using the appropriate Nikon 3.0 software and processed with Photoshop 4.0 software (Adobe Systems Inc., San Jose, California, USA).

### Analysis of the carotenoid content in wheat-*H*. *chilense* translocation lines

Carotenoids from mature grains were determined according to [29]. Grains of each line were milled to fine flour and 1 g of flour per replicate was extracted to analyze the carotenoid composition. Three biological replicates per line were analyzed. Briefly, samples were extracted in 4 mL acetone containing 0.1% BHT (butylated hydroxytoluene) by vortexing for 2 min and additionally sonicated for 5 min at room temperature. The mixture was centrifuged at 4500 rpm at 4°C for 10 min and the supernatant was recovered. The sediment was re-extracted with 4 mL of acetone until supernatant was colorless. Acetone extracts were pooled and dried under nitrogen stream. Dried extracts were stored at -25°C until HPLC analysis.

Composition of each sample was analyzed by HPLC as described in [58] by using a Waters liquid chromatography system equipped with a 600E pump, a 2998 photodiode array detector, and the Empower software (Waters). A C30 carotenoid column (250 x 4.6 mm, 5 μm) coupled to a C30 guard column (20 x 4.0 mm, 5 μm; YMC Europe GmbH, Germany) was used. Samples were prepared for HPLC by dissolving the dried carotenoid extracts in methanol: acetone (1:1 v:v). A ternary (methanol, water and methyl tert-butyl ether) gradient elution was used for carotenoid separation as is described in [58]. The flow rate was 1 mL min^-1^, column temperature was set to 25°C and the injection volume was 20 μL. The photodiode array detector was set to scan from 250 to 540 nm, and for each elution a Maxplot chromatogram, which plots each carotenoid peak at its corresponding maximum absorbance wavelength, was obtained. Carotenoids were identified by their retention time, absorption and fine spectra [59–62]. The carotenoid peaks were integrated at their individual maxima wavelength and their content were calculated using calibration curves of lutein (Sigma, St. Louis, MO, USA) for free and esterified lutein, and zeaxanthin (Extrasynthese). All operations were carried out on ice under dim light to prevent photodegradation, isomerizations and structural changes of the carotenoids.

### Statistical analysis

Statistical analyses were performed using STATISTIX 9.0 software (Analytical Software, Tallahassee, FL, USA). The analysis of variance (ANOVA) was based on randomised blocks. Means were separated using the Least Significant Difference (LSD) test with a probability level of 0.05.

### Protein extraction and quantification

Proteins were extracted following a phenol-based protocol described in [63] with slight modifications. Briefly, from each genotype two independent samples composed of a pool of 2–3 seeds was ground into a fine powder using a Star-Beater mill (VWR Company, Darmstadt, Germany). The ground tissue was resuspended in phenol extraction buffer (0.9 M sucrose, 0.5 M Tris-HCl, 50 mM EDTA, 0.1 M KCl, Milli-Q water and freshly added 1% Triton X-100, 2% β-mercaptoethanol and 1% protease inhibitor cocktail set VI (Merck KGaA, Darmstadt, Germany), pH 8) and homogenized on ice using Eppendorf micropestles. Samples were subsequently mixed with one volume of phenol solution equilibrated with 10 mM Tris HCl pH 8, 1 mM EDTA (Sigma, St. Louis, MO, USA), shaken for 1 min, incubated for 20 min in a tube rotator at 4°C and centrifuged at 18000 × *g* for 10 min at 4°C. The upper phenolic phase was collected and proteins were precipitated by adding five volumes of ice cold 0.1 M ammonium acetate and 13 mM DTT in methanol at -80°C for 2 h. A pellet of proteins was obtained by centrifugation at 20000 × *g* for 20 min at 4°C. Then, the pellet was washed once with ice cold 0.1 M ammonium acetate, 13 mM DTT in methanol and twice with 80% ice cold acetone. Finally, the pellet was air dried, dissolved in denaturing buffer (6 M urea, 50 mM ammonium bicarbonate pH 8) and stored at -80°C. Protein concentration was determined with the BCA Protein Assay Kit (Pierce Chemical Co., Rockford, EL), using BSA as a standard according to manufacturer’s instructions for the microplate procedure. Protein quality was checked by 1D-SDS-PAGE using Mini-Protean cell (Bio-Rad Laboratories, Richmond, CA) and 12% Mini-PROTEAN TGX precast polyacrylamide gels (Bio-Rad Laboratories, Richmond, CA) stained with Coomassie Blue G250.

### Reverse phase-liquid chromatography RP-LC-MS/MS analysis

Protein extracts in 6 M urea and 50 mM ammonium bicarbonate pH 8 were reduced and alkylated. Disulfide bonds from cysteinyl residues were reduced with 10 mM DTT for 1 h at 37°C, and then thiol groups were alkylated with 50 mM iodoacetamide for 1 h at room temperature in the dark. Samples were diluted to reduce urea concentrations below 1.4 M and digested using sequencing grade trypsin (Promega, Madison, WI) overnight at 37°C in a trypsin/protein ratio of 1:5 (w/w). Digestion was stopped by the addition of 1% TFA. Then, the supernatants were dried down and desalted onto ZipTip C18 Pipette tips (EMD Millipore Corporation, Billerica, MA) until mass spectrometric analysis.

Desalted digested proteins were dried out, resuspended in 0.1% formic acid and analyzed by RP-LC-MS/MS in an Easy-nLC II system coupled to an ion trap LTQ-Orbitrap-Velos-Pro mass spectrometer (Thermo Fisher Scientific Inc., Waltham, MA). The peptides were concentrated (on-line) by reverse phase chromatography using a 0.1 mm × 20 mm C18 RP precolumn (Acclaim PepMap100 nanoViper, Dionex), and then separated using a 0.075 mm × 100 mm C18 RP column (Acclaim PepMap100 nanoViper, Dionex) operating at 0.3 μl/min. Peptides from a 5 μg aliquot of the protein extract were eluted in a 180-min gradient of 5 to 40% solvent B (solvent A: 0.1% formic acid in water, solvent B: 0.1% formic acid, 80% acetonitrile in water). ESI ionization was carried out using a Nano-bore emitters Stainless Steel ID 30 μm (Proxeon) interface. The Orbitrap resolution was set at 30.000. Peptides were detected in survey scans from 400 to 1600 amu (1 μscan), followed by twenty data dependent MS/MS scans (Top 20), using an isolation width of 2 u (in mass-to-charge ratio units), normalized collision energy of 35%, and dynamic exclusion mode applied during 30 s periods. Peptide identification from raw data was carried out using the SEQUEST algorithm (Proteome Discoverer 1.4, Thermo Scientific). Database search was performed against Uniprot_Viridiplantae. The following constraints were used for the searches: tryptic cleavage after Arg and Lys, up to two missed cleavage sites, and tolerances of 10 ppm for precursor ions and 0.8 Da for MS/MS fragment ions. Searches were performed allowing optional Met oxidation and Cys carbamidomethylation. Search against decoy database (integrated decoy approach) was performed using false discovery rate (FDR) < 0.01. Protein identification by nLC-MS/MS was carried out at the CBMSO protein chemistry facility, a member of ProteoRed network.

### Bioinformatics and functional analysis of identified proteins

The output accessions obtained with the Proteome Discoverer software were exported to Microsoft Excel for data analysis. Firstly, a table containing information of all the proteins identified in the four genotypes analyzed was generated (S1 Table). The data obtained from the Uniprot-Viridiplantae search revealed that there were 372 proteins whose best hit was a protein with unknown function, meaning 50% of the proteins identified. Hence, to improve the information about the peptides matching proteins with unknown function a manual blastp was carried out. This analysis consisted on the blastp of the protein with unknown function with the Uniprot database; this allowed the identification of highly homologous proteins with an assigned function (identity with the protein with the best hit and the protein with described function > 80%).

In addition, a table containing the proteins exclusively identified in the genotypes with increased carotenoid content was created (Table 1). To this end, only the proteins that were present in the two replicates of each line were considered for the comparison between lines. Exceptionally, interesting proteins that were not exclusively found in one of the lines or in the two replicates of the proteomics experiments were also included in the list because they could have a relationship with the accumulation of carotenoids. These exceptions are indicated in the table and marked with asterisks.

**Table 1:** List of proteins exclusively identified in the protein extracts of the lines with enhanced carotenoid accumulation. Unless otherwise stated the proteins were identified in the two replicates of the lines and not in any other protein extracts. The Uniprot identification number (ID; http://www.uniprot.org/) and the protein name of the best matches of the identified peptides are included. When the best match corresponded to a protein with a yet unassigned function the protein with the highest homology (>80% identity) was also indicated in brackets. The proteins with a possible implication in carotenoid enrichment are highlighted in bold.

Line	Uniprot ID	Protein name
**Addition** 7**H** ^**ch**^	**O49996**	**14-3-3-like protein**
	F2CX17	Predicted protein (88% identity with cold shock domain protein 2; Q75QN9)
	W5FAI9	Uncharacterized protein (100% identity with defensin; A0A060AQ78)
	R7W8W0	Defensin-like protein 1
	**A5A8U9**	**26.4kDa heat-shock protein** *
7**H** ^**ch**^α·7**A**L	Q2QLR2	Glycine-rich RNA-binding protein GRP1A
	I1QDX3	Uncharacterized protein (99% identity with 2,3-bisphosphoglycerate-independent phosphoglycerate mutase; Q10LY9)
	F2E2F1	Predicted protein (100% identity with 60S ribosomal protein L21-2; M8CY06)
	F2CSZ7	Predicted protein
7**A**S·7**H** ^**ch**^β	**D2E9R6**	**Hsp organizing protein/stress-inducible protein**
	M7YCT7	3-ketoacyl-CoA thiolase 2, peroxisomal
	A2YP75	Putative uncharacterized protein
	W5A1H5	Uncharacterized protein
7**H** ^**ch**^α**·7A**L and 7**A**S·7**H** ^**ch**^β	Q39782	Alcohol dehydrogenase 2a
Addition 7**H** ^**ch**^ and7**H** ^**ch**^α·7**A**L	F2D712	Predicted protein
	W5GCI3	Uncharacterized protein
**Addition 7H** ^**ch**^ **and**7**H** ^**ch**^α**·7A**L and 7**A**S·7**H** ^**ch**^β	B9VUV5	Low molecular weight glutenin subunit
	Q1ZZT4	Low-molecular-weight glutenin subunit
	Q6J162	S-type low molecular weight glutenin
	K4AAT0	Uncharacterized protein (83% identity with Serpin-ZXA; Q75H81)
	M8BX24	Uncharacterized protein

* This protein was identified in the two replicates of addition 7**H** ^**ch**^ and also in one replicate of the line 7**A**S·7**H** ^**ch**^β.

## Results

### Development of wheat- chromosome 7H^ch^ translocation lines in hexaploid wheat

**Fig 2:**
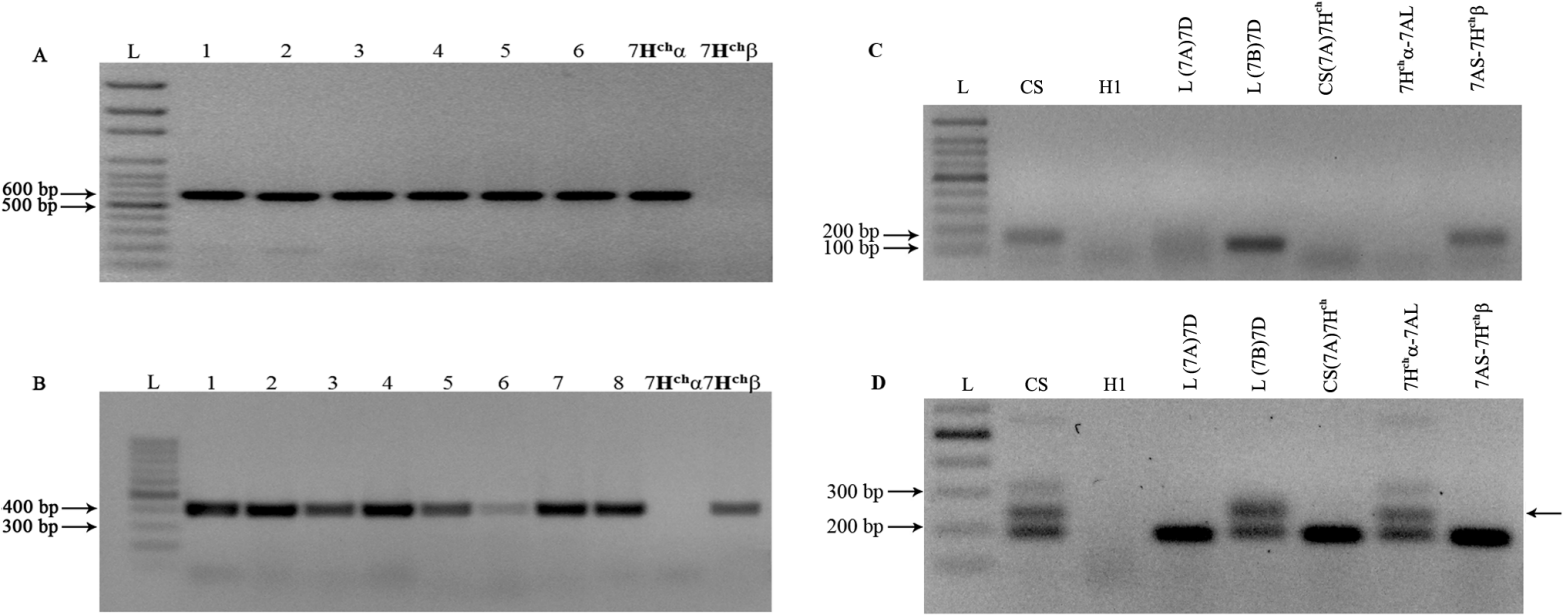
Identification of 7H^ch^α or 7H^ch^β chromosome arms in the wheat background and characterization of the wheat chromosome involved in chromosome translocations. The presence of A) 7**H** ^**ch**^α, B) 7**H** ^**ch**^β, C) 7**A**S and D) 7**A**L chromosome arms is detected using BAWU550, BAWU763, Xgwm471 and Xgwm332 markers, respectively. Positive controls 7**H** ^**ch**^α and 7**H** ^**ch**^β in panels A) and B) represent the wheat lines carrying either the 7**H** ^**ch**^α or the 7**H** ^**ch**^β telosomic chromosomes in the wheat background. Lanes 1–6 in A) and 1–8 in B) corresponds to several 7**H** ^**ch**^α∙7**A**L and 7**A**S∙7 **H** ^**ch**^β translocation lines, respectively. The polymorphic band in D) has been arrowed. L, ladder; CS, *T*. *aestivum* cv. Chinese Spring; H1, *H*. *chilense*; L(7**A**)7**D**, *T*. *turgidum* cv. Langdon (LDN) in which a pair of chromosome 7**A** has been substituted by chromosome 7**D** from CS; L(7**B**)7**D**, *T*. *turgidum* cv. Langdon (LDN) in which a pair of chromosome 7**B** has been substituted by chromosome 7**D** from CS; CS(7**A**)7**H** ^**ch**^, *T*. *aestivum* cv. Chinese Spring (CS) in which a pair of chromosome 7**A** has been substituted by a pair of chromosome 7**H** ^**ch**^ from *H*. *chilense*, 7**H** ^**ch**^α∙7**A**L and 7**A**S∙7**H** ^**ch**^β, disomic translocation lines in wheat.

**Fig 3:**
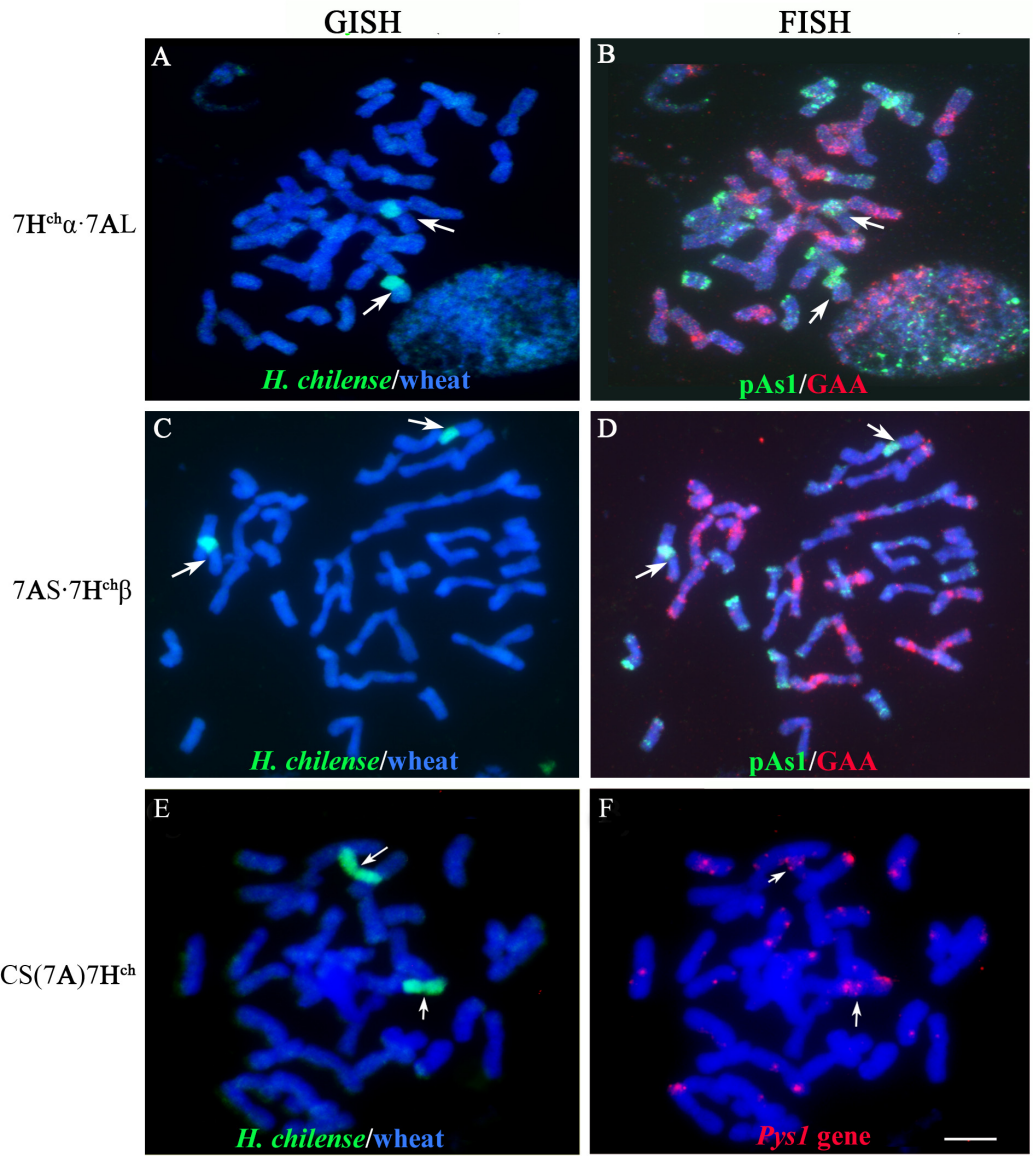
Example of *H*. *chilense* chromosome introgressions in the progeny derived from the crosses (7A)7H^ch^ substitution lines x *ph1b* mutant*2 and physical location of the *Psy1* gene on *H*. *chilense* chromosome 7H^ch^ of bread wheat CS-*H*. *chilense* (7A)7H^ch^ substitution line. Genomic *in situ* hybridization (GISH) was carried out using total *H*. *chilense* genomic DNA as a probe (detected in green). In fluorescence *in situ* hybridization (FISH) experiments, the pAs1 and the GAA sequences were used as probes (detected in green and red, respectively) to identify chromosomes involves in *H*. *chilense*-wheat translocations. A PCR amplification product (2538bp) of the *Psy1* gene was used as a probe for physical mapping of the *Psy1* locus. The DNA was counterstained with DAPI (blue). A**)** GISH and B) FISH pattern of a mitotic metaphase carrying two copies of the 7**H** ^**ch**^α∙7**A**L Robertsonian translocation (arrowed). C) GISH and D) FISH of a mitotic metaphase carrying two copies of 7AS∙7**H** ^**ch**^β Robertsonian translocation (arrowed). E) GISH and F) FISH of a (7**A**)7**H** ^**ch**^ substitution line showing two positive signals corresponding to the *Psy1* locus only on the two *H*. *chilense* chromosomes (arrowed). Scale Bar in F represents 10μm in all panels.

Crosses between chromosome 7**H** ^**ch**^ substitution line in wheat and the *ph1b* mutant in hexaploid wheat were made with the aim to introgress chromosome 7**H** ^**ch**^ in the background of the wheat *ph1b* mutant, to promote interespecific chromosome associations between chromosome 7**H** ^**ch**^ and its 7A wheat homoeologous and to reduce the size of chromosome 7**H** ^**ch**^ in the wheat background (Fig 1) [22]. Screening and characterization of plants carrying introgressions from *H*. *chilense* chromosome 7**H** ^**ch**^ were carried out by molecular markers and multicolor *in situ* hybridization. BAWU550 and BAWU763 microsatellites were used to identify several plants carrying chromosome 7**H** ^**ch**^ introgressions (Fig 2). The presence of both molecular markers indicated the presence of the whole chromosome 7**H** ^**ch**^ but could not discern between a whole chromosome introgression or heterozygous Robertsonian translocations between the *H*. *chilense* and the wheat homoeologous chromosomes, carrying one copy of 7**H** ^**ch**^α-7**A**L translocation and one copy of 7**A**S-7**H** ^**ch**^β translocation. Translocations between chromosome 7**H** ^**ch**^ and wheat chromosomes were detected by GISH (Fig 3). The use of molecular markers combined with GISH and FISH experiments enabled the determination of the exact chromosomal compositions and resolution of the chromosome arms involved in wheat-chromosome 7**H** ^**ch**^ translocations (Figs 2 and 3). Heterozygous 7**H** ^**ch**^α·7**A**L and 7**A**S·7**H** ^**ch**^β robertsonian translocations were only detected by *in situ* hybridization. Several homozygous 7**H** ^**ch**^α·7**A**L and 7**A**S·7**H** ^**ch**^β translocation lines were obtained in the final selfed population.

In addition, the physical localization of *Psy1* gene was performed by fluorescence *in situ* hybridization using a 2538bp fragment of the *Pys1* genomic DNA sequence as a probe. Based on the *Psy1* DNA sequence, primers were designed as described in the materials and methods section, to amplify the 2538bp fragment of the *Psy1* gene in *H*. *chilense*. As expected, the *Psy1* locus was visualized on *H*. *chilense* chromosome 7**H** ^**ch**^and no signals were detected on the homoeologous wheat chromosomes (Fig 3).

### Analysis of the carotenoid composition in wheat-*H*. *chilense* translocation lines

The carotenoid profile was determined in *H*. *chilense* translocation lines for chromosome 7**H** ^**ch**^ in wheat and compared to wheat. The main carotenoids identified in all samples were lutein (free and esterified with fatty acids) and zeaxanthin, accounting for more than 95% of the total carotenoids. Trace amounts of β-carotene were additionally detected in some of the samples but were below the quantification threshold. Quantification of individual carotenoids and the amount of total carotenoids are showed in Table 2. Total carotenoids (1215 ± 13 ng g^-1^ dry weight (DW)) content in the 7**H** ^**ch**^α∙7**A**L translocation line was double than the wheat control and similar to that of the 7**A**S∙7**H** ^**ch**^β translocation line (1133 ± 68 ng g^-1^ DW). As expected, the carotenoid content of the bread flour was the lowest (603 ± 59 ng g^-1^ DW), followed by the chromosome 7**H** ^**ch**^ addition line in bread wheat (803 ± 58 ng g^-1^ DW). Thus, the maximum carotenoid content was detected in the translocation lines for both 7**H** ^**ch**^α or 7**H** ^**ch**^β chromosome arms in the background of *ph1b* mutant. The maximum content for zeaxanthin was detected in the 7**A**S∙7**H** ^**ch**^β translocation line, although this line showed the minimum content in esterified lutein. Our results clearly indicate that the new translocation lines generated showed higher carotenoid content than both bread wheat and the wheat line carrying the addition of a pair of the whole chromosome 7**H** ^**ch**^, mainly due to the higher accumulation of free lutein.

**Table 2:** Carotenoid content in bread wheat, wheat-7H^ch^ addition lines, and *H*. *chilense*-wheat translocation lines. Data are mean ± SE of three biological replicates. The letters in italics indicate statistical significance (P < 0.05).

Wheat lines	Total carotenoids ng g^-1^ DW	Free lutein		Esterified lutein		Zeathantin	
		ng g^-1^ DW	%	ng g^-1^ DW	%	ng g^-1^ DW	%
Bread wheat	603 ± 59*c*	321 ± 39*b*	53.23	70 ± 13*c*	11.60	213 ± 6*ab*	35.32
Wheat-7**H** ^**ch**^ disomic addition	803 ± 58*b*	326 ± 17*b*	40.60	268 ± 25*a*	33.37	209 ± 15*b*	26.02
7**H** ^**ch**^α∙7**A**L disomic translocation	1215 ± 13*a*	844 ± 17*a*	69.47	176 ± 12*b*	14.47	195 ± 14*c*	16.08
7**A**S∙7**H** ^**ch**^β disomic translocation	1133 ± 68*a*	874 ± 43*a*	77.10	23± 5*d*	2.10	235 ± 30*a*	20.80

*ng per g of dry weight (DW): ng g^-1^ of dry weight

### Comparison of the seed proteomic profile among wheat and the introgression lines with carotenoid-enriched seeds

Seed proteins were extracted from bread wheat and the three introgression lines with carotenoid enriched-seeds: the wheat lines carrying the addition of chromosome 7**H** ^**ch**^, the translocation of 7**H** ^**ch**^α chromosome arm (7**H** ^**ch**^α∙7**A**L translocation) and the translocation of 7**H** ^**ch**^β chromosome arm (7**A**S∙7**H** ^**ch**^β translocation). The protein extraction protocol consisted on the extraction from two replicates of each line with a phenol-based buffer followed by precipitation with ammonium acetate. The quality and the complexity of the extracted proteins were checked by sodium dodecyl sulfate polyacrylamide gel electrophoresis (1D-SDS-PAGE) prior to Nano-scale liquid chromatographic tandem mass spectrometry (nLC-MS/MS). The band pattern of the seed extracts was highly similar among lines (S1 Fig). Then, a high sensitive system of reverse-phase nLC coupled to a high resolution and mass accuracy mass spectrometer (LTQ-Orbitrap-Velos-Pro) was used to analyse the samples. To minimise the number of false positives or misidentifications a high level of confidence was applied for protein identification. Thus, only peptides with 5 to 30 amino acids and a minimum of two peptides per protein allowed a positive identification. A false discovery rate (FDR) < 0.01 was also set. As a result, 741 different proteins were identified from all the seed extracts analysed (S1 Table).

**Fig 4:**
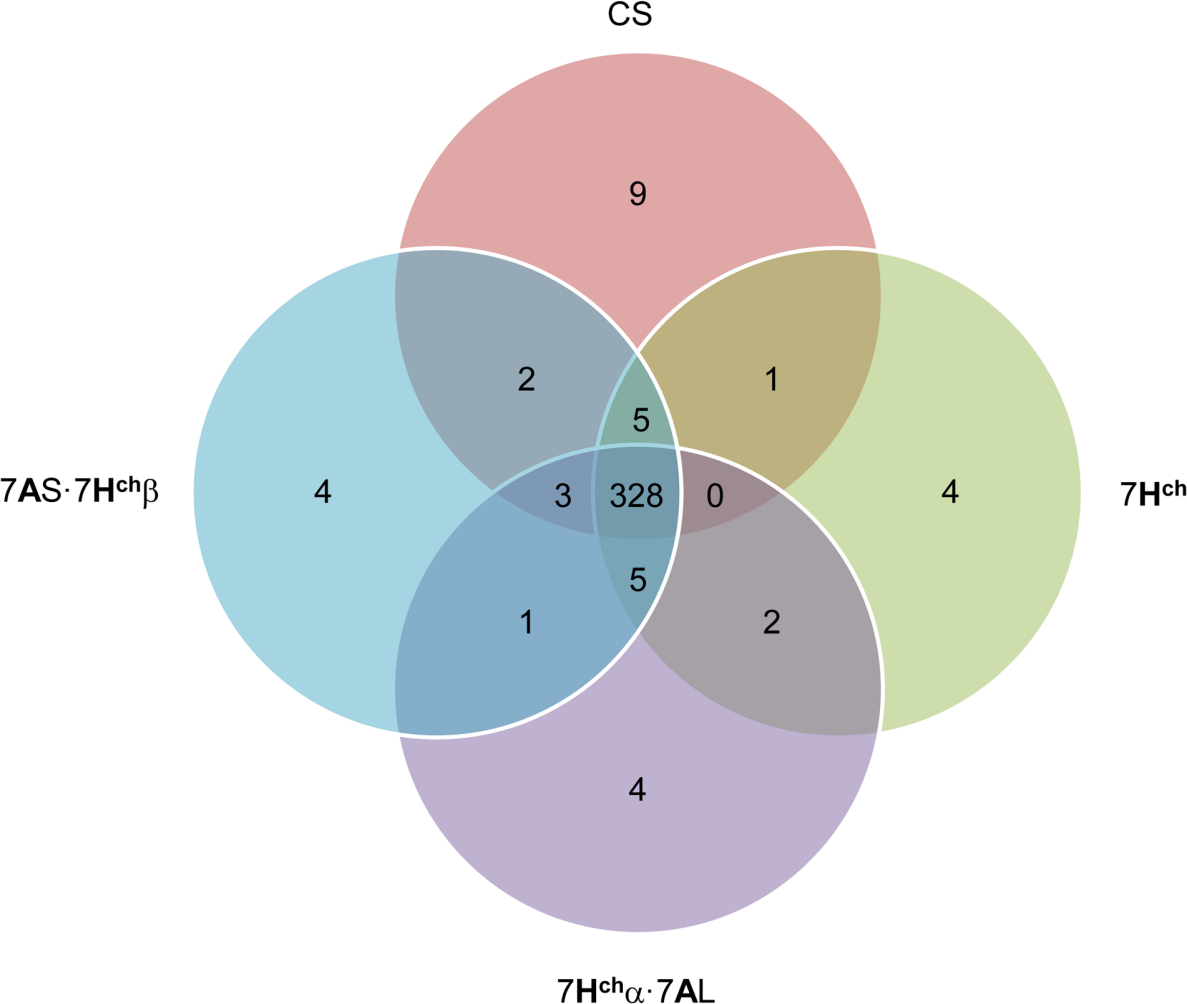
Venn diagram summarizing the proteins identified in seed extracts of bread wheat and carotenoid-enriched lines. Only peptides with 5 to 30 amino acids and a minimum of two peptides per protein allowed positive identifications, and peptide FDR < 0.01

Only proteins that were present in the two replicates of each line were considered for further analysis (368). Ninety percent of the proteins (328) were common to the bread wheat and the introgression lines, while only ten percent of the proteins (40) were specifically present in some of the lines (Fig 4). Twelve proteins were only identified in either the addition or in each of the translocation lines but not in the wild type. Out of them 7 proteins had as best hit a protein with unknown function, therefore to increase the information about these proteins manual blastp were performed in an attempt to find highly similar proteins with an assigned function. Only those proteins showing at least 80% identity were considered (S1 Table).

The search for proteins with functions that could be potentially related to the regulation of carotenoid accumulation was carried out by searching at the whole set of proteins that were not present in bread wheat but in some of the other lines with higher carotenoid contents. This analysis led to the selection of a 14-3-3 protein, a small heat shock protein (sHSP, 26.4 kDa), and a HSP70-HSP90 organizing protein (O49996, A5A8UA, and D2E9R6, respectively).

## Discussion

Most mapping studies in wheat agree that quantitative trait loci (QTL) located on group 7 chromosomes largely determine the yellow pigment content of the grains (YPC). The *Psy1* gene, which encodes for the first reaction of the carotenoid biosynthetic pathway, was considered a candidate gene to explain the YPC of wheat grain since it maps to chromosomes 7**A** and 7**B** of durum and bread wheat [64].

*Tritordeums*, which are amphiploids obtained after chromosome doubling of the hybrid between diploid, tetraploid or hexaploid wheat and *Hordeum chilense*, have higher carotenoid pigment content than durum or bread wheat [32]. Analysis of the flour pigment content in wheat-*H*. *chilense* addition lines led to the conclusion that chromosome 7**H** ^**ch**^ from *H*. *chilense* confers the capacity to accumulate higher carotene concentration in seeds [2] Moreover, the *Psy1* gene is the only gene related with the carotenoid biosynthetic pathway physically mapped in *H*. *chilense* [33]. Taking into account all this information, we developed crosses between the (7**A**)7**H** ^**ch**^ substitution line in wheat and the wheat *ph1b* mutant to facilitate chromosome associations and recombination between chromosome 7**H** ^**ch**^ and those from the wheat homoeologous group 7. Homozygous 7**H** ^**ch**^α·7**A**L and 7**A**S∙7**H** ^**ch**^β translocation lines in hexaploid wheat were obtained and the evaluation of the pigment content in this translocation lines was carried out. The 7**H** ^**ch**^α∙7**A**L translocation lines showed higher carotenoid content than bread wheat as expected because *Psy1* gene is located in 7**H** ^**ch**^α chromosome arm from *H*. *chilense* [30]. This *Psy1* locus has been cytogenetically mapped on chromosome 7**H** ^**ch**^ in a (7**A**)7**H** ^**ch**^ substitution line in bread wheat using the biotinyl tyramide system (Tyr-FISH) (Fig 3), and seemed to be specific from *H*. *chilense* as no signals were detected in any of the related wheat chromosomes 7**A**, 7**B** or 7**D**. In addition, the 7**A**S∙7**H** ^**ch**^β translocation line also showed higher total carotenoid content than the wheat control and similar to the 7**H** ^**ch**^α∙7**A**L. The high carotenoid levels in the 7**A**S∙7**H** ^**ch**^β line can be related to the presence of a QTL in the distal part of the 7**H** ^**ch**^β chromosome arm associated with the increment of YPC, although so far, there are no candidate genes described in this region related to YPC [30].

The proteomics analysis comparing the endosperm proteome of the addition of 7**H** ^**ch**^ and the translocation of the 7**H** ^**ch**^β chromosome arms revealed the presence of 14-3-3 and heat shock proteins (HSPs, Table 1). Both 14-3-3 and HSPs were previously described to be required for the translocation of nucleus-encoded chloroplast precursor proteins into the chloroplast [65]. For example, plant DXP reductoisomerase (DXR) which catalyses the second step in the MEP pathway has an N-terminal transit domain with a putative motif for a 14-3-3 binding site [66]. Therefore, the post-translational modifications of biosynthetic proteins due to the interaction with 14-3-3 proteins and/or HSPs could be involved in the accumulation of carotenoids observed in the introgressed lines (Table 2). Furthermore, several studies in tomato and grapefruit have revealed that HSPs are related to carotenoid accumulation [67–69]. The 26.4 KDa heat-shock protein (A5A8U9), which was present in the line with the addition of 7**H** ^**ch**^ could also be playing a key role in the accumulation of carotenoids as small heat shock proteins were found to be the most abundant proteins present in the carotenoid-protein complexes of cassava roots, suggesting their involvement in the accumulation of these pigments. [70].

## Conclusions

The translocation lines developed in this work are an important tool to enrich the carotenoid content in bread wheat. Moreover, there are not available neither substitution/addition lines nor translocation lines for chromosome 7**H** ^**ch**^ in durum wheat. Thus, these translocation lines are also a useful tool to transfer these chromosome arms into durum wheat, and therefore, to enrich carotenoid content in durum wheat.

The comparison of the proteomic profile of the wheat introgression lines with bread wheat CS revealed that the overall protein content was scarcely altered by the introgression of *H*. *chilense* chromosome 7**H** ^**ch**^ or 7**H** ^**ch**^α and 7**H** ^**ch**^β chromosome arms and suggested that HSPs and a 14-3-3-like protein could play a key role in the enhancement of carotenoid accumulation in seeds.

## Supporting Information

S1 Fig SDS-PAGE of seed protein extracts from bread wheat and carotenoid-enriched lines. SDS-PAGE stained with Coomassie Brilliant Blue G250 of the seed protein extracts obtained from bread wheat (CS, lane 1), wheat-7**H** ^**ch**^ disomic addition line (lane 2), and the 7**H** ^**ch**^α·7**A**L (lane 3) and 7**A**S·7**H** ^**ch**^β (lane 4) disomic translocation line**s.**
(TIF)

S1 Table Proteins identified by nLC-MS/MS. Proteins identified in seeds of bread wheat, wheat-7**H** ^**ch**^ disomic addition line, and the 7**H** ^**ch**^α·7**A**L and 7**A**S·7**H** ^**ch**^β disomic translocation lines analyzed by nLC-MS/MS. Uniprot accession number, description, sequence of identified peptides, number of amino acids (AAs) of the identified protein, molecular weight of the identified protein, and number of peptides identified in each line are described.
(XLSX)
