# Supplementary figures and images for: Novel Bread Wheat Lines Enriched in Carotenoids Carrying Hordeum chilense Chromosome Arms in the ph1b Background
Source: PLoS One. 2015 Aug 4;10(8):e0134598. doi: 10.1371/journal.pone.0134598 (PMC4524710; doi:10.1371/journal.pone.0134598)

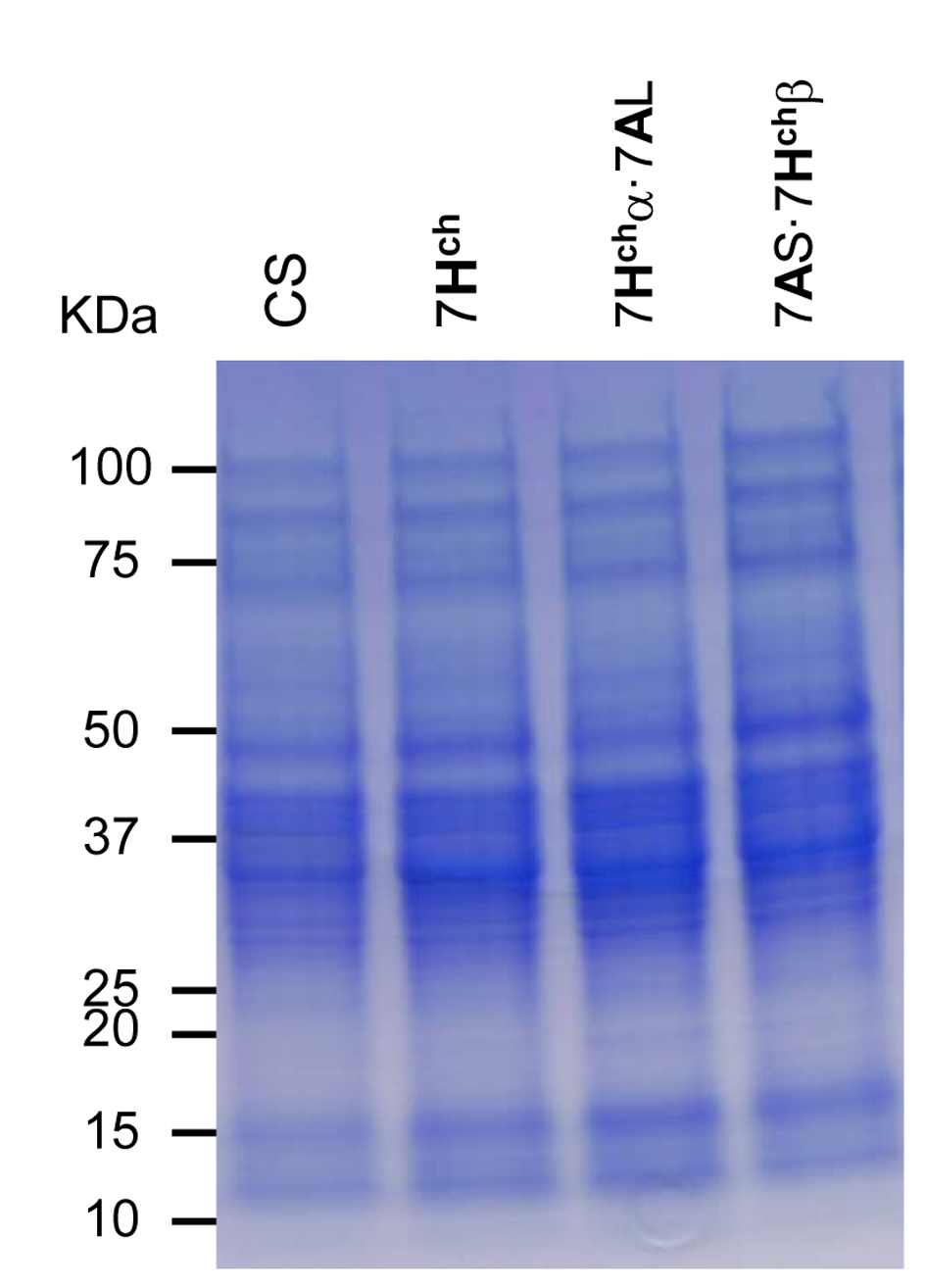

Supplement: S1 Fig — SDS-PAGE stained with Coomassie Brilliant Blue G250 of the seed protein extracts obtained from bread wheat (CS, lane 1), wheat-7H ch disomic addition line (lane 2), and the 7H chα·7AL (lane 3) and 7AS·7H chβ (lane 4) disomic translocation lines. (TIF) [file pone.0134598.s001.tif]
